# The structural brain network topology of episodic memory

**DOI:** 10.1371/journal.pone.0270592

**Published:** 2022-06-24

**Authors:** Melanie A. Matyi, Jeffrey M. Spielberg

**Affiliations:** Department of Psychological and Brain Sciences, University of Delaware, Newark, Delaware, United States of America; Kochi University of Technology, JAPAN

## Abstract

Episodic memory is supported by a distributed network of brain regions, and this complex network of regions does not operate in isolation. To date, neuroscience research in this area has typically focused on the activation levels in specific regions or pairwise connectivity between such regions. However, research has yet to investigate how the complex interactions of structural brain networks influence episodic memory abilities. We applied graph theory methods to diffusion-based anatomical networks in order to examine the structural architecture of the medial temporal lobe needed to support effective episodic memory functioning. We examined the relationship between performance on tests of verbal and non-verbal episodic memory with node strength, which indexes how well connected a brain region is in the network. Findings mapped onto the Posterior Medial memory system, subserved by the parahippocampal cortex and overlapped with findings of previous studies of episodic memory employing different methodologies. This expands our current understanding by providing independent evidence for the importance of identified regions and suggesting the particular manner in which these regions support episodic memory.

## Introduction

Converging evidence from both animal and human work suggests that episodic memory is instantiated in the brain via a network of regions centered on the medial temporal lobe (MTL) [1, 2], including the hippocampus and the parahippocampal, perirhinal, and entorhinal cortices [1]. In addition, retrosplenial cortex is highly interconnected with MTL regions, and strong evidence supports its critical role in episodic memory [3]. The prevailing model of episodic memory involves two parallel pathways, each subserving different aspects of memory and each facilitating communication between hippocampus and largely distinct sets of cortical association areas [1, 4, 5]. In both pathways, the hippocampus is a point of convergence for different types of contextual information about stimuli [2, 5]. The first pathway is referred to as the posterior medial (PM) memory system, operates via retrosplenial and parahippocampal cortices, and facilitates the flow of spatial information about the context of a stimulus [6]. The second pathway is referred to as the anterior temporal (AT) memory system, operates via perirhinal cortex, and facilitates the flow of non-spatial information about the attributes of a stimulus [6]. Functional connectivity analyses centered on retrosplenial/parahippocampal cortex and perirhinal cortex reveal that each are functionally correlated to distinct sets of neocortical association areas [5].

Although much is known about the specific brain regions supporting episodic memory, far less is known about how the *connectivity level of these regions with the larger brain network* supports episodic memory. Typical approaches involve examination of pairwise coupling (either structural or functional) with relevant regions (e.g., hippocampus). However, this approach does not capture the complex interactions that occur due to information exchange with multiple brain regions. Therefore, examination of only individual paths will provide a fundamentally incomplete picture. In contrast, methods like graph theory take the organization of brain networks into account and can index the importance of a brain region for a particular aspect of network processing [7]. For example, graph theory methods can quantify the extent to which brain regions are well connected in the network and, thus, facilitate evaluation of how this connectivity contributes to their influence on cognitive processes like episodic memory.

Recently, a number of studies have employed graph theory methods to examine how functional networks relate to memory. For example, such methods have been used to provide evidence for the importance of hippocampus and its role in functional networks supporting episodic memory [8, 9]. In particular, right hippocampus exhibited greater centrality (i.e., network importance) and communication efficiency during retrieval of vivid versus dim visual memories [9], whereas left hippocampus exhibited significant reorganization of its connectivity profile between remembered and forgotten conditions [8]. The functional networks supporting spatial and temporal memory retrieval have also been examined with these tools, finding increased connectivity during successful memory retrieval [10]. Lastly, graph theory methods have been used to examine functional network reorganization of the fronto-parietal network during episodic memory with low and high cognitive control demands [11]. Results revealed that cognitive control demands were related to differences in the level of network reconfiguration that occurred between memory encoding and item recognition conditions. Although this work has provided key insights into the network processes subserving episodic memory, most of the extant work has examined only networks derived from functional covariation during a task or at rest.

Key complementary information can be provided by the examination of structural networks based on white matter connectivity. Specifically, functional networks are constrained by structural networks [12], and thus, structural networks reflect the *capability* of the network to communicate. In addition, white matter networks will be more stable than the functional networks recruited on a given day [13], and thus may better reflect stable individual differences in episodic memory ability. Furthermore, evidence suggests that changes in the microstructure (i.e., fractional anisotropy) of white matter tracts, particularly the fornix, are associated with changes in episodic memory [14]. Therefore, the application of graph theory to structural networks is likely crucial to developing a comprehensive understanding of the brain basis of episodic memory. Support for this assertion is evident in a study applying graph theory methods to both functional and structural (derived from diffusion-weighted imaging) networks and using repetitive transcranial magnetic stimulation (rTMS) to investigate the impact of prefrontal cortex (PFC) connectivity on episodic memory encoding [15]. Results revealed that the impact of rTMS on functional connectivity depended on the level of structural connectivity, with high structural connectivity associated with a larger magnitude of impact of rTMS on functional connectivity. Although this study demonstrates the importance of examining structural networks for understanding memory, it focused primarily on PFC rather than the main regions supporting episodic memory, namely the hippocampus and parahippocampal cortex. Thus, the structural network topology of the regions central to episodic memory remains largely unexamined.

In the present study, we attempted to fill this gap in the literature by focusing on regions known to be critical to episodic memory (i.e., MTL and retrosplenial cortex). Specifically, we applied graph theory methods to structural brain networks (derived from diffusion-weighted imaging) in order to elucidate how the level of structural connectivity in these nodes support episodic memory. In particular, we examined node strength, which indexes first-order connectivity of brain regions, thus reflecting the connectivity strength of a given brain region [16]. This property was chosen, because it is easily interpretable, has demonstrated utility in characterizing network structure, and is appropriate to use with weighted networks. Additionally, node strength is a fundamental property that greatly influences many other network measures (e.g., clustering coefficient) [7], and thus, we chose to examine node strength to establish the basic connectivity profile of MTL regions. An advantage of node strength over other metrics with similar interpretations (e.g., node degree), in the context of weighted networks, is that link weights are incorporated into the computation of node strength. Measures such as node degree require binarization of the network, which requires the selection of a link threshold (e.g., set links with weights above that threshold to 1, and below the threshold to 0). In the case of the networks used in the present study, the selection of that threshold is arbitrary, and very different results can emerge when different thresholds are used [17]. Thus, node strength is a less biased metric when using weighted networks than node degree. We focused specifically on retrosplenial cortex and MTL regions due to their core role in facilitating episodic memory. Although neocortical regions contribute to episodic memory, they do so in arguably less central ways as compared to MTL regions [18, 19] and are typically recruited under particular conditions. For example, regions of PFC facilitate the recall of information in the presence of interference or distraction [4]. Therefore, it is not clear that one would expect the structural connectivity strength of neocortical regions to relate to episodic memory ability outside of these conditions, and thus the focus of the present work is on the MTL/retrosplenial cortex. For completeness, we have included supplemental analyses examining neocortical regions identified using Neurosynth with the key term ‘episodic memory’ (see S1 and S2 Tables).

We examined how the connectivity strength of MTL regions related to performance on both verbal and non-verbal measures of episodic memory via a series of robust regression analyses. Both verbal and non-verbal measures of episodic memory were examined, because different connectivity patterns may support different encoding and retrieval modalities. This idea is supported by work indicating that the left and right hemispheres are more active during verbal and non-verbal tasks of episodic memory, respectively [20]. Additionally, memory was tested in different ways in each task: in the verbal task, participants identified previously presented stimuli and rejected foils, whereas in the non-verbal task, participants ordered stimuli in the order in which they were presented. Thus, different regions may support accurate performance on these two tasks. For example, perirhinal cortex of the AT memory system is typically not involved in episodic memory, but is associated with remembering the temporal order of stimuli, and thus, may support performance on tasks requiring this information [21]. Regions in the MTL were examined, including the hippocampus, entorhinal cortex, three parahippocampal areas (components of the parahippocampal cortex), presubiculum, and perirhinal cortex [1]. Additionally, the retrosplenial cortex was examined, as it and the parahippocampal cortex are proposed to be key regions supporting episodic memory in the PM memory system [6].

In summary, there is currently no clear understanding of how episodic memory emerges from the structural connections of the MTL regions. Thus, we examined how the node strength of each MTL region and retrosplenial cortex is associated with performance on two tasks that index episodic memory in order to gain insight into the network mechanisms supporting such memory. We predicted that better performance on both episodic memory tests would be associated with greater node strength for hippocampus, entorhinal cortex, parahippocampal areas, retrosplenial cortex, and presubiculum. In addition, we predicted that better performance on the non-verbal episodic memory test, in particular, would be associated with greater perirhinal cortex node strength, given evidence that this region is active during tasks requiring memorization and recall of the temporal order of stimuli.

## Materials and methods

### Participant data

We used data collected from 1,053 healthy participants [M age = 28.75, SD = 3.68; female = 571 (54.28%); White = 798 (75.86%), Black = 148 (14.06%), Asian/Pacific = 63 (5.98%), American Indian/Alaskan = 2 (0.19%), Multiple = 26 (2.47%), Not reported = 15 (1.43%), Hispanic/Latino = 88 (8.37%)] as part of the Human Connectome Project (HCP). Briefly, the HCP offers a database of anonymous structural, diffusion, and functional MRI for research purposes [22]. We conducted secondary analysis on de-identified, open-access data after agreeing to the HCP Open Access Data Use Terms. Written informed consent, including consent to share de-identified data, was acquired by the HCP and approved by the Washington University institutional review board. One subject was excluded because connectivity could not be computed for a number of ROIs as no streamlines were detected during tractography. Eleven participants did not complete all tasks and were excluded, leading to a final n = 1,041.

### Cognitive measures

Along with the memory tests focused on in the present work, measures of crystalized knowledge and processing speed (see below) were used as covariates of no interest to ensure that findings were not driven by variance related to these processes.

#### Verbal episodic memory

Verbal episodic memory was indexed by the Penn Word Memory Test (IWRD), which is part of the Penn Computerized Cognitive Battery. The task has excellent reliability, as evidenced by the high internal consistency of the test, and construct validity, as demonstrated by strong associations with well-established measures of episodic memory [23, 24]. Participants were presented with a list of 20 words and instructed to memorize them for a later test. Following the presentation of the target words, participants were iteratively shown 40 words, which included the 20 target words mixed with the 20 new words (i.e., foil). Target and foil words were matched for memory-related characteristics, including length, frequency, concreteness, and imagery using Paivio’s norms [24, 25]. Participants decided whether they had previously seen the word by choosing “definitely yes,” “probably yes,” “probably no,” or “definitely no.” Credit was given for each correctly identified target word (“definitely yes” and “probably yes” responses) and correctly rejected foil (“definitely no” and “probably no” responses; maximum score = 40) [24].

#### Non-verbal episodic memory

Non-verbal episodic memory was indexed by the Picture Sequence Memory Test (PicSeq), which is part of the NIH Toolbox. The task has excellent test-retest reliability and construct validity, as demonstrated by its strong associations with well-established measures of episodic memory in addition to weak associations with measures of other cognitive abilities [26]. Participants were presented with sequences of pictures depicting objects and activities in a particular order and, simultaneously, the pictures were verbally described. Following each presentation of a 15-picture (trial 1) or 18-picture (trial 2) sequence, the pictures from the given trial were randomized and shown to participants all at once. Participants had to reconstruct the order of the pictures by placing them in order. Credit was given for each correctly placed adjacent pair of pictures (e.g., if pictures 6 and 7 are placed in order, a point was earned, regardless of where in the sequence these pictures were placed) (maximum score = 31).

#### Crystallized cognition

The Crystallized Cognition Composite score is the combination of the two NIH Toolbox tasks measuring crystallized cognition, the Picture Vocabulary Test and Reading Test. The scores for these two tests were normalized and averaged together to calculate the composite score. Crystallized cognition reflects abilities that are highly dependent on past learning and are consistent across the lifespan. The Picture Vocabulary Test measures general vocabulary knowledge. Participants were presented with four images and, simultaneously, an audio recording of a word. Participants then had to select the image that best matched the meaning of the word. The Reading Test measures reading decoding skills. Participants were asked to read and pronounce letters and words as accurately as possible. Higher scores on the vocabulary and reading tests indicate higher vocabulary and better reading abilities, respectively.

#### Processing speed

Processing speed, a measure of fluid ability, was indexed by the Pattern Comparison Processing Test, part of the NIH Toolbox. Speed of processing measures how much time it takes an individual to complete a task. In the Pattern Comparison Processing Test, participants were shown two side-by-side pictures and had to decide if they pictures were the same or not. Higher scores indicate faster task completion.

For all NIH Toolbox tasks (i.e., non-verbal episodic memory, crystallized cognition and processing speed), scores were scaled by normalizing them across the NIH Toolbox Normative Sample. Thus, a score of 100 on these tasks indicates performance that was at the national average and a score of 115 or 85, indicates performance 1 SD above or below the national average [27]. Additionally, although age-adjusted scaled scores are available for these measures, unadjusted scaled scores were used, as the IWRD score is not age-adjusted, and thus, age was included as a covariate in all analyses.

### Data acquisition

Data were acquired on a modified 3T Skyra System (Siemens) using a 32-channel coil. A T1-weighted structural image was acquired (TR = 2400ms; TE = 2.14ms; TI = 1000ms; flip angle = 8°; voxel size = .7x.7x.7mm) [28]. Diffusion acquisition involved a spin-echo EPI sequence [29, 30] with multiband EPI [31, 32] and 270 diffusion-weighted directions (TR = 5520ms; TE = 89.5ms; flip angle = 78°; refocusing flip angle = 160°; voxel size = 1.25x1.25x1.25mm; multiband factor = 3; b-values = 1000, 2000, 3000 s/mm^2^) [28, 33].

### HCP MRI preprocessing

All imaging data passed HCP quality assurance [34] and were run (by HCP) through several standardized preprocessing pipelines. The use of this (standardized) preprocessed data allows for greater methodological transparency and replicability across studies. Structural T1-weighted images first underwent gradient distortion and bias field correction. Next, T1 images were run through FreeSurfer’s standard pipeline to obtain a participant-specific subcortical segmentation, delineation of the cortical mantle, and segmentation of a white matter mask [35]. Diffusion data were run through an HCP pipeline in FSL to normalize b_0_ image intensity across runs and correct for EPI distortion, eddy-current induced distortions, gradient-nonlinearities, and subject motion [36, 37]. Next, diffusion data was processed in FSL’s bedpostx toolbox, which creates the files necessary for performing probabilistic tractography [38].

### Connectivity atlas

We used an atlas that included a 182-region (per hemisphere) cortical parcellation created by HCP using multi-modal imaging data [39], in conjunction with a 6-region (per hemisphere) subject-specific subcortical segmentation obtained via FreeSurfer [40]. The cortical atlas was warped to each participant’s cortical mantle using FreeSurfer transformations, then projected into 3d space. The HCP atlas and FreeSurfer segmentation each generated hippocampus ROIs, which were combined, thus resulting in a total of 186 nodes per hemisphere. Networks were calculated separately for right and left hemispheres, because evidence suggests that memory processes are lateralized, with the left hemisphere involved primarily in verbal memory and the right hemisphere involved in non-verbal memory [41, 42]. FreeSurfer ROIs representing white matter were combined to create a white matter mask for use in tractography.

### Creation of connectivity matrices

**Fig 1:**
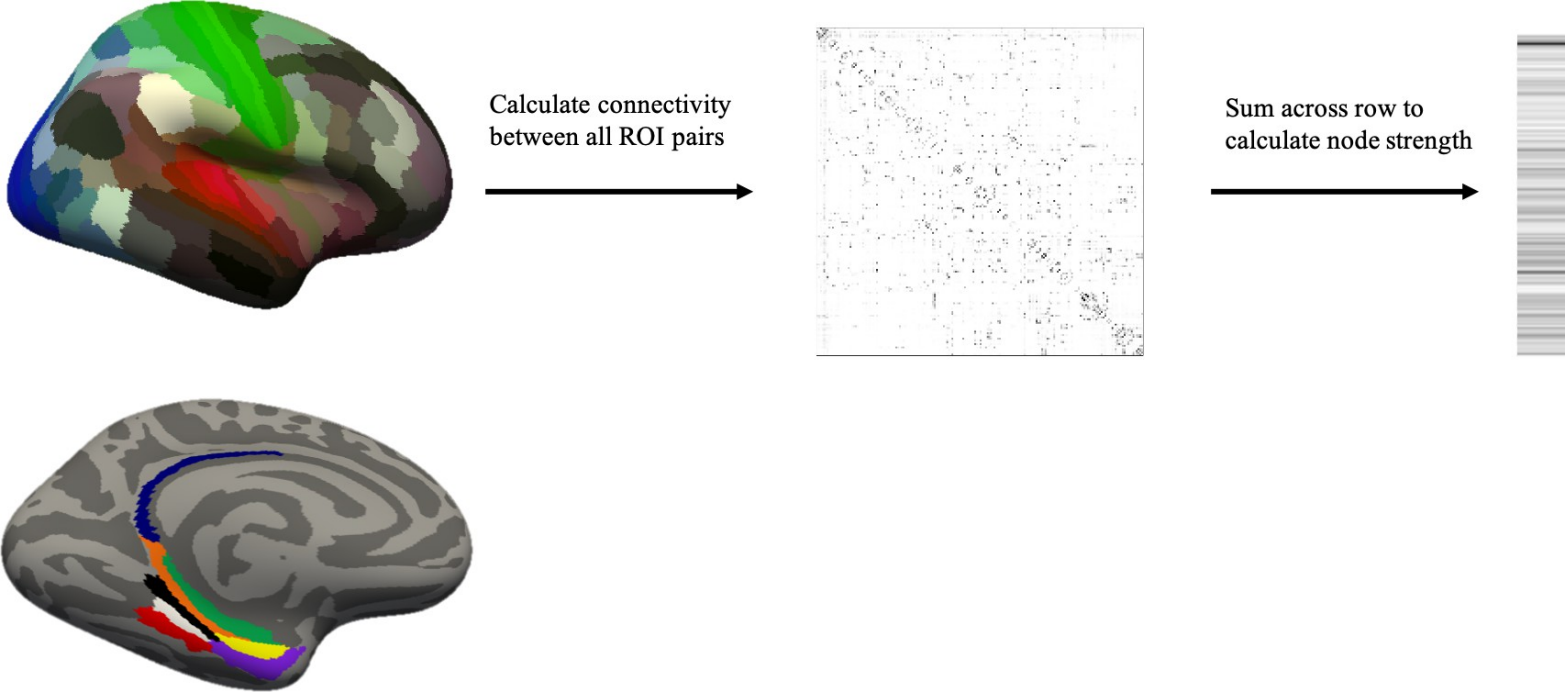
Overview of data processing. Top row: lateral view of the cortical HCP atlas (the subcortical ROIs are not visible in this view). Connectivity between all ROI pairs in the HCP-FreeSurfer atlas along with computed node strength are depicted. Bottom row: medial-inferior view of the 8 ROIs investigated shown. Retrosplenial cortex (blue), presubiculum (orange), parahippocampal area 1 (black), 2 (white) and 3 (red), perirhinal ectorhinal cortex (purple), entorhinal cortex (yellow), and hippocampus (green).

Interregional white matter connectivity was estimated using probabilistic tractography [43, 44] via FSL’s probtrackx2, which infers the orientation of a tract by repeatedly sampling from the principal diffusion direction calculated in bedpostx. A distribution of the tract’s path from each voxel using these estimates is then built. Multiple tracts are sampled from each voxel, and each propagation step is based on a randomly chosen orientation from the probability map. The estimated connectivity between two regions is equal to the probability of a tract starting at the seed region and going through the target region [45]. To obtain connectivity estimates from each ROI to every other ROI in the atlas, tractography was performed using the GPU version of probtrackx2 with the following options in addition to the compulsory arguments: ‘network’ (use network mode, which only retains paths that meet a different seed mask), ‘loopcheck’ (stop if path loops back on itself), ‘opd’ (output path distribution), ‘onewaycondition’ (apply waypoint condition to each half of tract separately), ‘waypoints = <white matter mask>‘ (paths must pass through white matter), ‘cthr = 0.2’ (curvature threshold), ‘nsteps = 2000’ (number of steps per sample), ‘steplength = 0.5’ (length of each step), ‘nsamples = 5000’ (total number of samples), ‘fibthresh = 0.01’ (threshold volume fraction to consider other fiber orientations), ‘distthresh = 0.0’ (discard samples shorter than 0.0mm), and ‘sampvox = 0.0’ (sample random points within a sphere with this radius in mm from the center of the seed voxel). These parameters ensured that 5,000 sample tracts were generated from the center of each voxel of each ROI and only tracts that (i) reached a target ROI and (ii) passed through white matter were retained. This resulted in a 186x186 connectivity matrix for each hemisphere, for each participant, where each entry represented the streamline count between each pair of nodes (see Fig 1). Importantly, streamline count covaries with both the number of axons connecting two regions and the microstructural integrity of those axons [46, 47]. The diagonal elements of the matrices represent self-connections and were excluded from analyses. Because 5,000 sample tracts are sent out from each voxel of each ROI, larger ROIs are oversampled. To account for variability related to differences in ROI size within and across individuals and differences in the ability of tractography to reconstruct different white matter pathways [48], the retained (i.e., not rejected by inclusion and exclusion criteria) streamline counts originating from each seed ROI were divided by the total number of tracts that were retained for that ROI [49]. Thus, each of the resulting values reflects the proportion of streamlines originating from the seed ROI that connects to each of the other ROIs. Due to the probabilistic nature of the tracking algorithm, and potential non-reciprocal connections between regions, the number of streamlines originating from region ‘A’ and terminating in region ‘B’ is not equivalent to the number originating from ‘B’ and terminating in ‘A’, causing the upper and lower diagonals of the initial connectivity matrix to be non-symmetric. However, because diffusion MRI (dMRI) cannot detect directionality, the number of tracts from seed region ‘A’ to target region ‘B’ should be equivalent to that from seed region ‘B’ to target region ‘A’. Thus, matrices were symmetrized by averaging the number of tracts of the two matrix elements representing the same connection.

### Graph theory metrics

Node strength, a measure of connectivity strength of individual nodes, was calculated for each node in the Graph Theory GLM (GTG) toolbox [50]. This graph property was chosen because it is easily interpretable, frequently used, and relatedly, has demonstrated utility in characterizing network structure [7]. Node strength is the sum of the weights of all connections to a given node [16]. For the current study, only eight ROIs were examined per hemisphere: hippocampus (including subiculum), presubiculum, entorhinal cortex, parahippocampal areas 1–3 (forming the posterior parahippocampal gyrus), perirhinal ectorhinal cortex and retrosplenial cortex.

### Data analysis

To identify nodes that support episodic memory processes, nodal graph metric values were entered as dependent variables in robust regression in the GTG toolbox [50], with episodic memory task score entered as the independent variable and age, percentage of the diffusion scan completed, subject motion (i.e., mean absolute framewise displacement), processing speed, and crystallized cognition score entered as covariates of no interest. Variables are defined in Table 1. Significance of the relationship between node strength and performance on the episodic memory tasks was determined via permutation tests (5,000 repetitions). In order to rein-in outliers, node strength was winsorized (within region) across participants to ±3 standard deviations. Analyses were also conducted without winsorized node strength values to evaluate the potential impact of outliers on results. False Discovery Rate (FDR) was used to correct for multiple comparisons across ROIs (8 per hemisphere), hemispheres, and tasks. Adjusted p-values were considered significant if less than 0.05.

**Table 1:** Variables.

*Dependent Variable*	*Covariates*	*Independent Variables*
Node strength	Age	IWRD
	Percentage of diffusion scan completed	PicSeq
	Processing speed	
	Crystallized cognition	

*Note*. IWRD = Penn Word Memory Test; PicSeq = NIH Toolbox Picture Sequence Memory Test.

## Results

### Behavioral results

Participants generally performed well on the IWRD, as indicated by a mean score of 35.65 out of a possible 40 total points, and on the PicSeq, as indicated by a mean of 112.00 (national average is 100; see Table 2).

**Table 2:** Descriptive statistics of episodic memory tests.

	N	Mean (SD)
IWRD	1041	35.65 (2.91)
PicSeq	1041	112.00 (13.27)

*Note*. IWRD = Penn Word Memory Test; PicSeq = NIH Toolbox Picture Sequence Memory Test; SD = standard deviation.

### Association of episodic memory system nodes with episodic memory test performance

**Fig 2:**
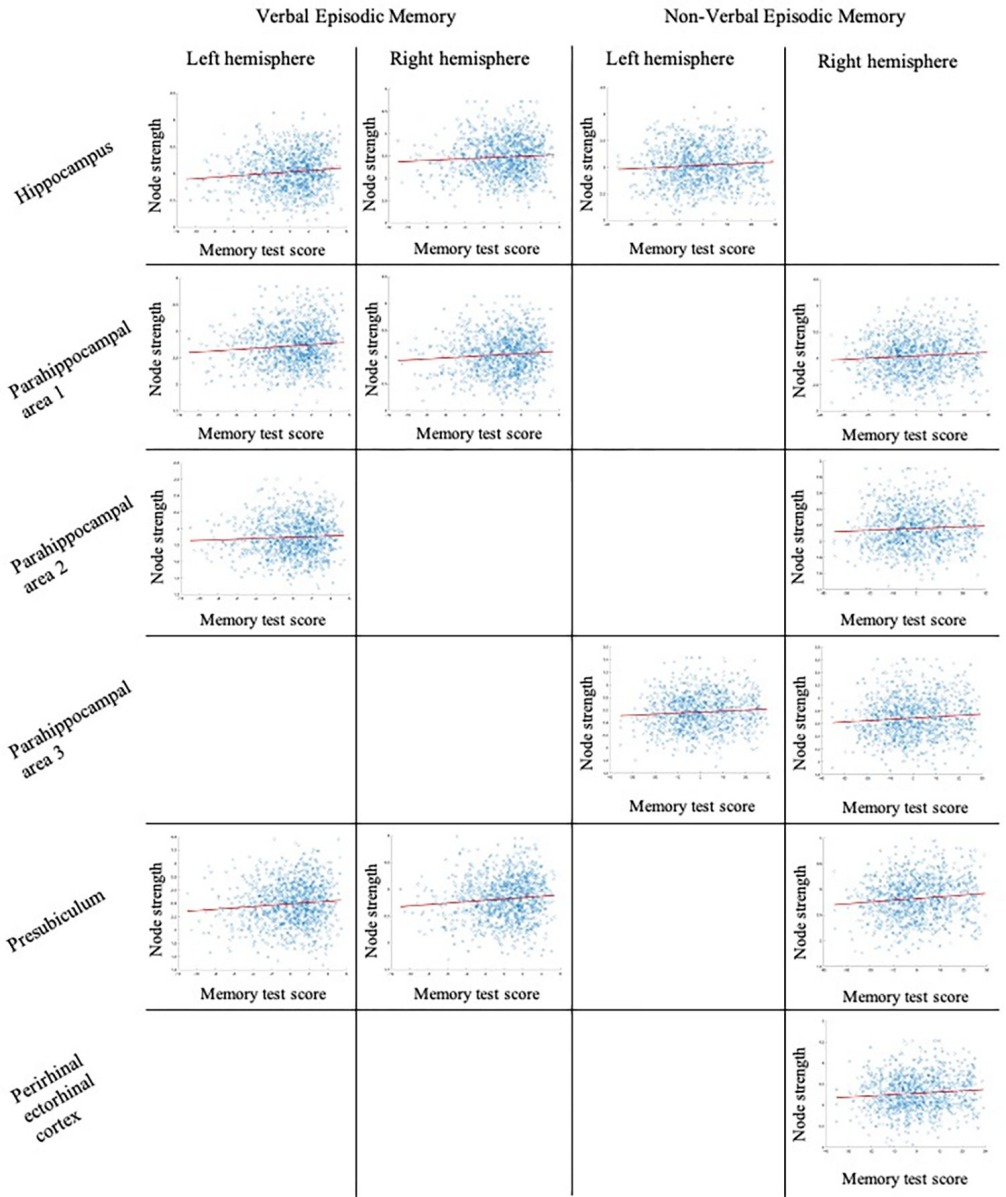
Winsorized node strength by residualized episodic memory test performance. Node strength values are winsorized to ±3 standard deviations. Episodic memory test values are the residualized scores after accounting for covariates.

In partial support of our hypothesis, better *verbal* episodic memory (indexed by higher IWRD scores) was associated with greater *node strength* for bilateral hippocampus, parahippocampal area 1, and presubiculum, and left parahippocampal area 2 (see Table 3; Fig 2). Better *non-verbal* episodic memory (indexed by higher PicSeq scores) was associated with greater *node strength* for bilateral parahippocampal area 3, left hippocampus and right perirhinal cortex, parahippocampal areas 1 and 2, and presubiculum (see Table 4). Results remained the same when analyses were conducted without reining-in node strength values, indicating that outliers did not affect results (see S3 and S4 Tables; S1 Fig).

**Table 3:** Brain regions’ node strength significantly associated with verbal episodic memory test performance.

Descriptive Name	HCP Name	beta	t-value	Uncorrected *p*-value	FDR-corrected *p*-value
Left hippocampus	n/a	.0150	3.91	< .001	< .001
Left parahippocampal area	PHA1	.0115	2.83	.004	.020
Left parahippocampal area	PHA2	.0057	2.36	.018	.041
Left presubiculum	PreS	.0122	3.53	< .001	.002
Right hippocampus	n/a	.0115	2.61	.011	.032
Right parahippocampal area	PHA1	.0120	3.03	.003	.015
Right presubiculum	PreS	.0159	3.73	< .001	.002

*Note*. Node strength values are winsorized to ±3 standard deviations. HCP Name = label from Human Connectome Project atlas.

**Table 4:** Brain regions’ node strength significantly associated with non-verbal episodic memory test performance.

Descriptive Name	HCP Name	beta	t-value	Uncorrected *p*-value	FDR-corrected *p*-value
Left hippocampus	n/a	.0021	2.55	.010	.032
Left parahippocampal area	PHA3	.0017	2.59	.009	.032
Right perirhinal ectorhinal cortex	PeEc	.0026	2.66	.006	.024
Right parahippocampal area	PHA1	.0022	2.52	.013	.035
Right parahippocampal area	PHA2	.0014	2.38	.016	.038
Right parahippocampal area	PHA3	.0021	2.89	.003	.015
Right presubiculum	PreS	.0033	3.53	< .001	.002

*Note*. Node strength values are winsorized to ±3 standard deviations. HCP Name = label from Human Connectome Project atlas.

## Discussion

The goal of the present study was to identify structural network nodes that support successful episodic memory. To date, associations between brain regions and episodic memory have been established largely using bivariate approaches examining functional or direct structural connectivity. However, the circuitry supporting episodic memory is complex, and thus, only limited insights into the set of connections that support episodic memory can be gained by examination of pairwise connections in isolation. Although a few studies have examined graph properties related to episodic memory, these investigations have been limited to functional networks. Thus, we aimed to examine how the *structural connectivity level* of medial temporal lobe (MTL) regions and retrosplenial cortex contribute to different types of episodic memory. To achieve this aim, we employed graph theory methods to test the relationship between structural connectivity node strength and performance on verbal and non-verbal episodic memory tests.

Importantly, because connectivity strength depends solely on a node’s placement within the network, the current study provides complementary and independent evidence of associations between the retrosplenial cortex and MTL regions and episodic memory. In particular, we index the importance of regions based upon the extent to which they are well-connected in the network. This approach can capture the complexity of the network by examining each region within the context of the entire network. Thus, compared to examining direct connectivity only, the methods employed in the present work provide insights into how the overall connectivity level of regions in the MTL support episodic memory abilities.

Dual systems models of the neural circuitry supporting episodic memory posit convergence of two pathways on the hippocampus, one mediated by the perirhinal cortex and the other by the parahippocampal cortex [51]. Two systems for memory-guided behavior have been proposed based on this model [6] and current results map onto this proposed organization. Briefly, the Posterior Medial (PM) system supports object-context associations and converges on the hippocampus via the parahippocampal cortex, and the Anterior Temporal (AT) system supports recognition memory and processing of non-spatial information and converges on the hippocampus via the perirhinal cortex. More specifically, evidence suggests that the role of perirhinal cortex in recognition memory is driven by familiarity (versus recollection) memory [21]. In the present study, brain regions with node strength significantly related to verbal episodic memory belong to the PM memory system, including the parahippocampal areas and presubiculum. Our finding that regions of the PM system are important for accurate verbal episodic memory, suggests that associations between the words and the context in which they are learned (i.e., learned in test for target words or learned elsewhere for foil words) may be supported by the PM system [6, 24].

Brain regions with node strength significantly related to non-verbal episodic memory were associated with both the AT and PM systems. Regions related to the PM system included parahippocampal areas and presubiculum. The processing of spatial context and forming object-context associations, supported by the PM system, is integral to accurate performance on the PicSeq task which requires participants to remember the order of pictures [26]. The perirhinal cortex, an important region in the AT system, was also identified as central to supporting non-verbal episodic memory. In similar, temporal order memory tasks, the perirhinal cortex responds to the order of stimulus presentation, suggesting that the perirhinal cortex is important in forming item-time associations [21]. Thus, both the AT and PM systems appear to be critical to performing well on the PicSeq task, which is achieved by remembering the order of presented images.

Overall, results were similar across both episodic memory tasks, with the exception of the perirhinal cortex for the PicSeq task. Connectivity strength of bilateral hippocampus, parahippocampal area, and presubiculum was associated with performance on both verbal and non-verbal tests of episodic memory, suggesting that the capability of these regions to influence the brain network is important regardless of the modality in which episodic memory is tested. This pattern of findings is consistent with previous research, implicating MTL regions generally in episodic memory processes [1]. The MTL is an area of convergence in which unimodal and polymodal sensory information is sent and integrated to facilitate memory processes. Thus, many inputs converge on the MTL, most of which are reciprocal [52, 53]. This organization implies that MTL regions should be strongly connected to facilitate memory processes. This is particularly true of the hippocampus which is, for most types of episodic memory, the final point of convergence. Specifically, information from both the parahippocampal cortex/PM system and perirhinal cortex/AT system is sent to hippocampus [6, 52, 54]. Therefore, it is likely that the connectivity strength of the hippocampus is critical to general episodic memory processes, as it integrates information from a variety of sources.

Present findings largely align with previous research investigating the neural correlates of verbal and non-verbal memory. Specifically, studies relating neural measures to IWRD performance have inconsistent results, some of which converge with those of the current study. Consistent with the present results, higher CBF in the parahippocampal cortex, among other non-MTL regions, has been linked to IWRD recall [55]. Interestingly, one study measuring functional activity during administration of the IWRD and another study associating brain region volume to IWRD performance found significant relationships with a number of regions, including frontal regions, angular gyrus, and temporal fusiform, but not in any MTL regions [56, 57]. For non-verbal episodic memory, there is more consistent support for the importance of MTL regions. Using a spatial memory test analogous to the IWRD, Roalf and colleagues (2014) also identified parahippocampal cortex as functionally related to memory performance. Similarly, hippocampal volume was significantly related to performance on an analogous task [56]. An additional study that utilized task fMRI data from a subset of 376 participants from the HCP dataset in a subsequent memory test paradigm found that activity in parahippocampal gyri during the encoding phase of a working memory task was associated with successful (vs. unsuccessful) recollection in the PicSeq task [58]. Thus, results across these studies at least partially converge with those of the present study, suggesting that parahippocampal area is particularly important to non-verbal episodic memory, as parahippocampal area emerged as significant across both functional and structural modalities and distinct but related memory paradigms.

### Strengths and limitations

The present study benefited from a large sample (n = 1,041), high-quality diffusion data which provided high-accuracy tractography, and the use of an ROI atlas created using multiple imaging modalities. Several limitations must be considered, including that the findings are dependent on the connectivity algorithms used, and thus we cannot exclude the possibility that systematic biases in these algorithms impacted our results. Additionally, because directionality cannot be inferred from dMRI, more fine-grained analyses integrating such directionality are not possible. Lastly, we only examined one graph metric, node strength, and thus it is possible that different regions may be found to be important to episodic memory ability were other metrics of structural network connectivity examined (e.g., degree centrality).

## Conclusion

The present study examined the structural network architecture supporting episodic memory. Using graph theory methods, we identified regions whose connectivity strength is associated with performance on episodic memory tests. The specific regions identified herein are consistent with proposed theories of episodic memory (e.g., greater reliance on the PM memory system) and lateralization of non-verbal memory processes in addition to overlap with previous studies using either the same or related memory tasks. Overall, similar regions emerged as related to successful episodic memory performance across hemispheres and task modality (verbal and non-verbal). Present findings expand our current understanding by showing that the structural connectivity of brain regions in the network is important to both verbal and non-verbal episodic memory.

The methodology employed in the present study examined the connectivity strength of each MTL brain region to determine their contribution to supporting verbal and non-verbal episodic memory performance. This provides complementary and independent evidence to support the role of these regions in episodic memory, as previously established by examination of pairwise coupling of regions and relating their functional activity to episodic memory performance. Our results suggest that examination of how regions are structurally positioned within the network and their resultant capacity to support functional processes is also important in understanding how episodic memory processes emerge. In summary, the findings presented herein advance our knowledge of how verbal and non-verbal episodic memory depend upon a complex network of brain regions.

## Supporting information

S1 Fig Node strength by residualized episodic memory test performance. Node strength includes outliers. Episodic memory test values are the residualized scores after accounting for covariates.
(TIF)

S1 Table Prefrontal cortex regions’ node strength significantly associated with verbal episodic memory test performance. Node strength values include outliers. HCP Name = label from Human Connectome Project atlas.
(DOCX)

S2 Table Prefrontal cortex regions’ node strength significantly associated with non-verbal episodic memory test performance. Node strength values include outliers. HCP Name = label from Human Connectome Project atlas.
(DOCX)

S3 Table Brain regions’ node strength significantly associated with verbal episodic memory test performance. Node strength values include outliers. HCP Name = label from Human Connectome Project atlas.
(DOCX)

S4 Table Brain regions’ node strength significantly associated with non-verbal episodic memory test performance. Node strength values include outliers. HCP Name = label from Human Connectome Project atlas.
(DOCX)
