# Supplementary figures and images for: The structural brain network topology of episodic memory
Source: PLoS One. 2022 Jun 24;17(6):e0270592. doi: 10.1371/journal.pone.0270592 (PMC9232126; doi:10.1371/journal.pone.0270592)

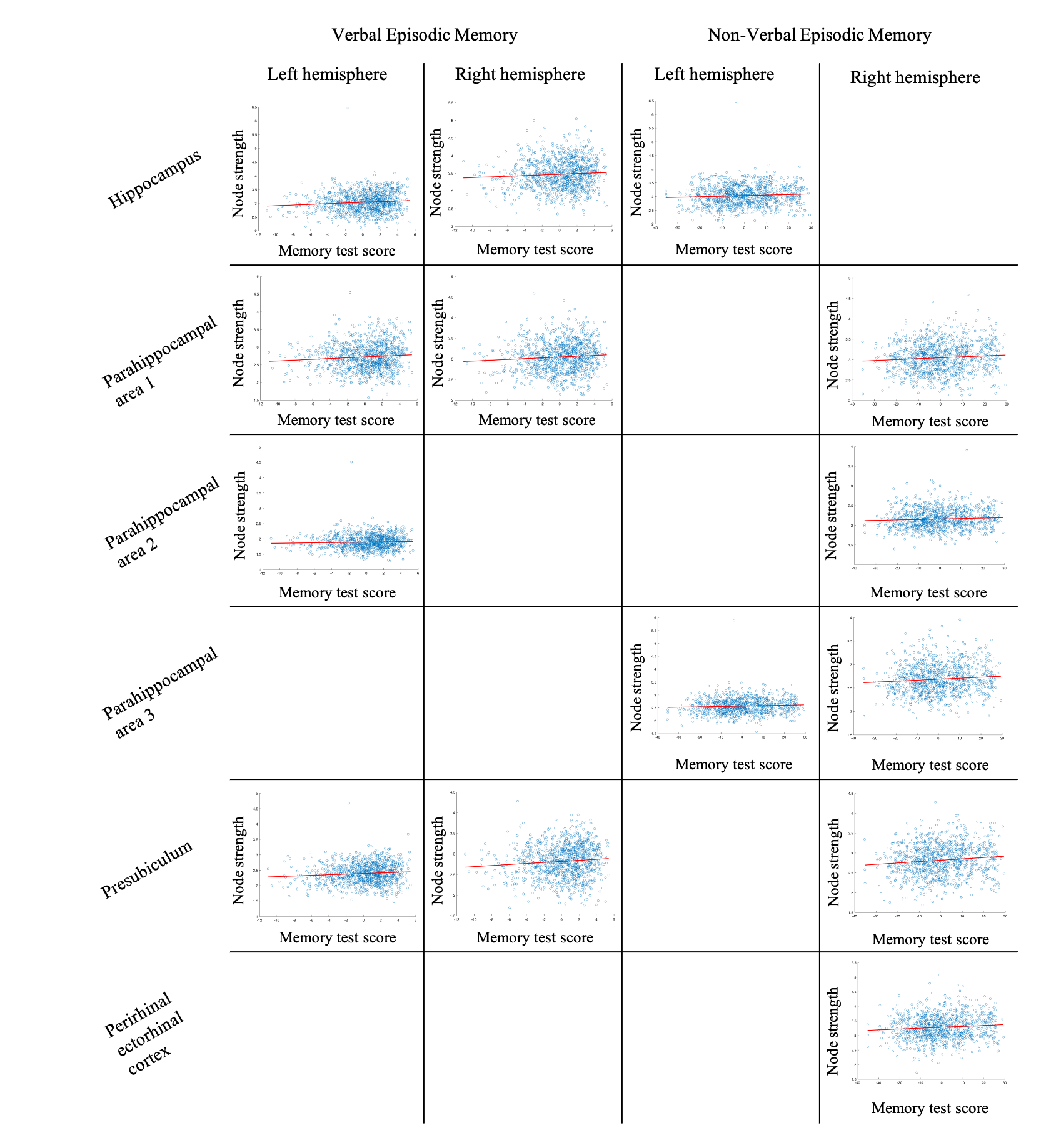

Supplement: S1 Fig — Node strength includes outliers. Episodic memory test values are the residualized scores after accounting for covariates. (TIF) [file pone.0270592.s001.tif]
