# Supplementary material for: The structural brain network topology of episodic memory
Source: PLoS One. 2022 Jun 24;17(6):e0270592. doi: 10.1371/journal.pone.0270592 (PMC9232126; doi:10.1371/journal.pone.0270592)
Supplement: S1 Table — Node strength values include outliers. HCP Name = label from Human Connectome Project atlas. (DOCX) [file pone.0270592.s002.docx]

**S1 Table. Prefrontal Cortex Regions’ Node Strength Significantly Associated with Verbal Episodic Memory Test Performance.**

| Descriptive Name | HCP Name | beta | t-statistic | FDR-corrected p-value |
| --- | --- | --- | --- | --- |
| Left dorsal BA 10 | 10d | -0.003 | -1.08 | 0.295 |
| Left ventral BA 8A | 8Av | -0.006 | -1.45 | 0.153 |
| Left lateral BA 8B | 8BL | 0.001 | 0.32 | 0.744 |
| Left anterior BA 9 | 9a | 0.001 | 0.54 | 0.582 |
| Left medial BA 9 | 9m | 0.002 | 0.67 | 0.506 |
| Left anterior BA 9-46 | a9-46v | 0.001 | 0.59 | 0.556 |
| Left posterior BA 9-46 | p9-46v | -0.001 | -0.26 | 0.797 |
| Right dorsal BA 10 | 10d | 0.001 | 0.27 | 0.786 |
| Right ventral BA 8A | 8Av | -0.001 | -0.23 | 0.826 |
| Right lateral BA 8B | 8BL | 0.000 | 0.11 | 0.914 |
| Right anterior BA 9 | 9a | -0.002 | -1.18 | 0.232 |
| Right medial BA 9 | 9m | 0.004 | 1.33 | 0.189 |
| Right anterior BA 9-46 | a9-46v | 0.001 | 0.65 | 0.521 |
| Right posterior BA 9-46 | p9-46v | 0.000 | 0.11 | 0.909 |

*Note.* Node strength values include outliers. HCP Name = label from Human Connectome Project atlas.
