# Supplementary material for: The structural brain network topology of episodic memory
Source: PLoS One. 2022 Jun 24;17(6):e0270592. doi: 10.1371/journal.pone.0270592 (PMC9232126; doi:10.1371/journal.pone.0270592)
Supplement: S2 Table — Node strength values include outliers. HCP Name = label from Human Connectome Project atlas. (DOCX) [file pone.0270592.s003.docx]

**S2 Table. Prefrontal Cortex Regions’ Node Strength Significantly Associated with Non-Verbal Episodic Memory Test Performance.**

| Descriptive Name | HCP Name | beta | t-statistic | FDR-corrected p-value |
| --- | --- | --- | --- | --- |
| Left dorsal BA 10 | 10d | 0.000 | 0.42 | 0.665 |
| Left ventral BA 8A | 8Av | 0.000 | -0.37 | 0.712 |
| Left lateral BA 8B | 8BL | 0.001 | 1.25 | 0.201 |
| Left anterior BA 9 | 9a | 0.001 | 2.16 | 0.032 |
| Left medial BA 9 | 9m | 0.000 | 0.01 | 0.994 |
| Left anterior BA 9-46 | a9-46v | 0.000 | 0.08 | 0.936 |
| Left posterior BA 9-46 | p9-46v | 0.001 | 1.02 | 0.301 |
| Right dorsal BA 10 | 10d | 0.000 | -0.26 | 0.789 |
| Right ventral BA 8A | 8Av | 0.002 | 1.70 | 0.087 |
| Right lateral BA 8B | 8BL | 0.000 | 0.73 | 0.468 |
| Right anterior BA 9 | 9a | 0.000 | -0.66 | 0.493 |
| Right medial BA 9 | 9m | 0.001 | 1.97 | 0.048 |
| Right anterior BA 9-46 | a9-46v | 0.000 | -0.18 | 0.863 |
| Right posterior BA 9-46 | p9-46v | 0.001 | 1.04 | 0.298 |

*Note.* Node strength values include outliers. HCP Name = label from Human Connectome Project atlas.
