# Supplementary material for: The structural brain network topology of episodic memory
Source: PLoS One. 2022 Jun 24;17(6):e0270592. doi: 10.1371/journal.pone.0270592 (PMC9232126; doi:10.1371/journal.pone.0270592)
Supplement: S3 Table — Node strength values include outliers. HCP Name = label from Human Connectome Project atlas. (DOCX) [file pone.0270592.s004.docx]

**S3 Table. Brain Regions’ Node Strength Significantly Associated with Verbal Episodic Memory Test Performance.**

| Descriptive Name | HCP Name | beta | t-statistic | FDR-corrected p-value | Uncorrected p-value |
| --- | --- | --- | --- | --- | --- |
| Left hippocampus | n/a | .015 | 3.94 | < .001 | < .001 |
| Left entorhinal cortex | EC | .002 | 0.43 | .710 | .666 |
| Left perirhinal cortex | PeEc | .007 | 1.74 | .110 | .079 |
| Left parahippocampal area | PHA1 | .012 | 2.86 | .021 | .005 |
| Left parahippocampal area | PHA2 | .006 | 2.38 | .044 | .019 |
| Left parahippocampal area | PHA3 | .005 | 1.83 | .104 | .068 |
| Left presubiculum | PreS | .012 | 3.51 | .003 | < .001 |
| Left retrosplenial cortex | RSC | .002 | 0.77 | .478 | .433 |
| Right hippocampus | n/a | .011 | 2.61 | .028 | .010 |
| Right entorhinal cortex | EC | .001 | 0.34 | .759 | .735 |
| Right perirhinal cortex | PeEc | .010 | 2.18 | .067 | .031 |
| Right parahippocampal area | PHA1 | .012 | 3.06 | .017 | .003 |
| Right parahippocampal area | PHA2 | .005 | 2.10 | .068 | .037 |
| Right parahippocampal area | PHA3 | .003 | 0.86 | .434 | .380 |
| Right presubiculum | PreS | .016 | 3.76 | < .001 | < .001 |
| Right retrosplenial cortex | RSC | .006 | 1.27 | .246 | .200 |

*Note.* Node strength values include outliers. HCP Name = label from Human Connectome Project atlas.
