# Supplementary material for: The structural brain network topology of episodic memory
Source: PLoS One. 2022 Jun 24;17(6):e0270592. doi: 10.1371/journal.pone.0270592 (PMC9232126; doi:10.1371/journal.pone.0270592)
Supplement: S4 Table — Node strength values include outliers. HCP Name = label from Human Connectome Project atlas. (DOCX) [file pone.0270592.s005.docx]

**S4 Table. Brain Regions’ Node Strength Significantly Associated with Non-Verbal Episodic Memory Test Performance.**

| Descriptive Name | HCP Name | beta | t-statistic | FDR-corrected p-value |
| --- | --- | --- | --- | --- |
| Left hippocampus | n/a | .002 | 2.57 | .028 |
| Left entorhinal cortex | EC | .001 | 1.75 | .110 |
| Left perirhinal cortex | PeEc | .002 | 2.01 | .073 |
| Left parahippocampal area | PHA1 | .002 | 2.07 | .068 |
| Left parahippocampal area | PHA2 | .001 | 0.95 | .420 |
| Left parahippocampal area | PHA3 | .002 | 2.62 | .028 |
| Left presubiculum | PreS | .002 | 2.09 | .068 |
| Left retrosplenial cortex | RSC | < .001 | 0.12 | .907 |
| Right hippocampus | n/a | .002 | 1.65 | .137 |
| Right entorhinal cortex | EC | .001 | 1.62 | .149 |
| Right perirhinal ectorhinal cortex | PeEc | .003 | 2.65 | .028 |
| Right parahippocampal area | PHA1 | .002 | 2.49 | .028 |
| Right parahippocampal area | PHA2 | .001 | 2.40 | .044 |
| Right parahippocampal area | PHA3 | .002 | 2.87 | .020 |
| Right presubiculum | PreS | .003 | 3.53 | .002 |
| Right retrosplenial cortex | RSC | .002 | 1.93 | .084 |

*Note.* Node strength values include outliers. HCP Name = label from Human Connectome Project atlas.
